# IL-1β is involved in docetaxel chemoresistance by regulating the formation of polyploid giant cancer cells in non-small cell lung cancer

**DOI:** 10.1038/s41598-023-39880-2

**Published:** 2023-08-07

**Authors:** Song Zhao, Sining Xing, Lili Wang, Mingyue Ouyang, Shuo Liu, Lingyan Sun, Huiying Yu

**Affiliations:** Laboratory of Basic Medicine, General Hospital of Northern Theatre Command, No. 83 Wenhua Road, Shenhe District, Shenyang, 110016 Liaoning China

**Keywords:** Lung cancer, Senescence, Mechanisms of disease, Cancer therapeutic resistance

## Abstract

Docetaxel (Doc) is a cornerstone of chemotherapy; however, treatment with Doc often and inevitably leads to drug resistance and the formation of polyploid giant cancer cells (PGCCs). In this study, we investigated the effect of Doc on non-small cell lung cancer to explore the role of PGCCs in drug resistance and the molecular mechanisms that regulate this resistance. We found that Doc induced G2/M cell cycle arrest and cell death in A549 and NCI-H1299 cells. However, many cells remained alive and became PGCCs by decreasing the expression of key regulatory proteins related to the cell cycle and proliferation. Notably, the PGCCs showed typical features of senescence, especially upregulation of p21 and p-histone H2A.X expression. Moreover, the mRNA level of IL-1β in the senescence-associated secretory phenotype was increased significantly with the development of PGCCs. Inhibition of IL-1β reduced the expression of p-histone H2A.X and promoted polyploidy to enhance the proapoptotic effect of Doc. Taken together, our results suggested that IL-1β was involved in the formation of PGCCs and regulated the senescence of PGCCs, which contributed to drug resistance to Doc. Therefore, targeting IL-1β in PGCCs may be a novel approach to overcome drug resistance.

## Introduction

Lung cancer is a common malignant tumour worldwide, among which non-small cell lung cancer (NSCLC) accounts for approximately 85% and is the leading cause of cancer-related mortality^1^. Chemotherapy is one of the traditional treatments and Doc is a cornerstone of chemotherapy for NSCLC^2^. In many cases, Doc prolongs overall and progression-free survival, but it also inevitably leads to drug resistance and relapse following chemotherapy^3,4^. Doc is a microtubule inhibiting agent that causes cell cycle arrest in mitosis, after which the cells either die in mitosis or aberrantly exit (mitotic slippage) and survive as polyploid cells^5^. Polyploidy is a conserved mechanism in stress responses, and multiple treatment stresses, including chemotherapeutic drugs, can induce polyploidization of tumour cells^6^, which leads to the formation of PGCCs. PGCCs are believed to be highly related to therapy resistance and tumour repopulation after therapy^7,8^. Although PGCCs were described over a century ago and increasingly recognized, the mechanisms underlying the formation of PGCCs and their role in drug resistance have not yet been clearly clarified.

Cellular senescence is usually defined as a cellular state of cell cycle arrest and proliferative inhibition^9^. This process is increasingly recognized as an important concept in cancer biology. Chemotherapy can induce cellular senescence, which is a different fate than cell death and may be related to PGCCs^10^. In fact, PGCCs are always accompanied by a senescent phenotype, including nondividing hypertrophic cell morphology, cell cycle arrest, and enhanced β-galactosidase activity^11^. Moreover, PGCCs constitute a transiently senescent subpopulation of cancer cells that arise in response to chemotherapy^12^. Although senescent cells lose the ability to proliferate, they remain alive and retain metabolic activity^10^. Hence, PGCCs can escape the fate of death through a special mechanism in which they acquire a senescent phenotype.

Senescent cells undergo metabolic changes and secrete a series of active factors, which are called the senescence-associated secretory phenotype (SASP)^13^. Inflammatory cytokines in the SASP are thought to be critical for cancer development and the acquisition of drug resistance^14^. IL-1β is an important inflammatory cytokine, and anticancer treatments are able to promote IL-1β production^15^. Recent studies have shown that IL-1β can be directly produced by tumour cells, which lead to treatment failure^16^. Although IL-1β is often related to drug resistance, the role of IL-1β in drug resistance is still not clearly clarified.

In the current study, we investigated the effect of Doc on the formation and senescence of PGCCs in NSCLC, focusing on the role of inflammatory factors in these processes. Our study showed that Doc induced G2/M cell cycle arrest and promoted the formation of PGCCs. On this basis, we found that PGCCs showed typical features of senescence and that IL-1β in the SASP increased significantly with the development of PGCCs. Notably, inhibition of IL-1β reduced the expression of p-histone H2A.X (γ-H2A.X) and promoted polyploidy to enhance the proapoptotic effect of Doc. Our data suggested that targeting IL-1β in PGCCs might be a promising strategy for reversing chemoresistance to Doc.

## Materials and methods

### Reagents and antibodies

All reagents and primary antibodies used in this study are listed in the Supplementary material (Additional file 1 for Tables S1 and S2). All stock solutions were stored at − 20 °C before use.

### Cell lines and treatment schedule

The human NSCLC cell lines A549 and NCI-H1299 were obtained from the American Type Culture Collection (ATCC, Manassas, VA, USA). Cells were cultured in DMEM/F-12 medium (Shanghai Basalmedia Technologies Co., Ltd.) with 10% foetal bovine serum (FBS; VivaCell) and maintained in the presence of 5% CO_2_ at 37 °C. For Doc treatment, cells were treated with Doc at 100 nM for 24 h (h) and then allowed to recover in regular medium for three days. Cells were divided into three groups: control group, Doc (24 h) group and Doc (24 h) + 3 days group. For diacerein treatment, cells were treated with diacerein at 1 μM for 24 h to inhibit IL-1β before treatment with Doc. Cells were divided into 4 groups: control group, Dia group, Doc group and Doc + Dia group. DMSO was added to the culture system as vehicle control.

### Cell labelling and microscopic imaging analysis

Dil (Thermo Fisher Scientific; Waltham, MA, USA) and Hoechst 33342 (Beyotime; Shanghai, China) dye were used to label the cell membrane and nucleus. Cells were seeded in 24-well culture plates (ABC Biochemistry; Hong Kong, China) and treated with Doc. Then, the cells were rinsed once in PBS and fixed in 4% paraformaldehyde for 20 min (min). After incubation with Dil (5 μM) and Hoechst 33342 (1:100) for 30 min at 37 °C, the morphology of the cells was visualized under a Ti-S inverted fluorescence microscope (Nikon, Japan) with NIS-Elements D 3.2 software.

### Cell imaging and cell count

The morphology of the cells was imaged under a conventional light microscopy (Nikon, Japan). After recording images, all tangible cells (attached/viable cells and floating cells) were collected in the culture system, and the number of cells was counted in a hemocytometer chamber under a microscope.

### Cell cycle and DNA content analysis

Cells were harvested, washed once with ice-cold PBS and fixed in 80% iced methanol overnight at − 20 °C. Then, the cells were resuspended in 500 μl of PBS containing PI (50 μg/ml) and incubated in the dark for 30 min at room temperature. Cell cycle and DNA content analysis were carried out by using a FACS Canto® Π flow cytometer (BD Canto; San Jose, CA, USA). Cell cycle distribution was analysed by FlowJo 7.6 Software and DNA content was analysed by BD FACSDiva Software.

### Analysis of cell death

The effect of Doc on the apoptosis of A549 and NCI-H1299 cells was quantitated using the 7-AAD/PE Annexin-V apoptosis detection kit (BD Biosciences; San Jose, CA, USA) according to the manufacturer’s instructions. The cells were collected and resuspended at a concentration of 1 × 10^6^ cells/mL in 1 ml of binding buffer (1 ×). The cell suspension (100 μL) was incubated with 5 μL of Annexin V (PE) and 5 μL of 7-AAD for 15 min at room temperature in the dark. Then, 1 × binding buffer (400 μL) was added. PE Annexin-V or 7-AAD fluorescent intensities were analysed by a FACS Canto® Π flow cytometer and 10,000 cells were evaluated in each sample.

### Senescence-associated β-galactosidase (SA-β-Gal) staining

Cells were plated on coverslips in 24-well culture plates and then treated with Doc. Following the instructions provided by the manufacturer, an SA-β-Gal staining kit (Beyotime; Shanghai, China) was used to detect the cells attached to the coverslips. The development of blue colour in cells was imaged using a Ti-S inverted microscope.

### Western blot analysis of protein levels

Protein extraction and immunoblotting were performed^17^. Briefly, cells were collected and washed twice with precooled PBS. Total protein was extracted by using RIPA lysis buffer containing protease and phosphatase inhibitor cocktails (Roche; Basel, Switzerland). The concentration of proteins was quantified by using a Pierce BCA protein assay kit (Thermo Fisher Scientific). Equal amounts of protein sample (about 30 μg) were used for western blot analysis. β-Actin was used as an internal control.

### Xenograft tumour model

Six-week-old male BALB/c-nude mice were provided by Changsheng Biotechnology Co., Ltd. (Liaoning, China). The human NSCLC A549 cells (5 × 10^5^ cells) suspended in 100 μl of PBS were subcutaneously injected into the right flank of nude mice and allowed to establish tumours. When the tumour volume reached approximately 100 mm^3^, tumour-bearing mice were randomized into two groups: PBS group and Doc group, with 5 mice in each group, which were intraperitoneally injected with PBS or Doc (10 mg/kg) once every 7 days for a total of 4 times. After 4 weeks, the mice were euthanized by cervical dislocation following the American Veterinary Medical Association (AVMA) Guidelines for the Euthanasia of Animals (2020). The tumours were surgically removed and photographed. All of the animal experiments were performed following the approval of the Institutional Animal Care and Use Committee (IACUC) of Liaoning Changsheng Biotechnology Co., Ltd. (Approval No. CSE202108002). The study is reported in accordance with ARRIVE guidelines (https://arriveguidelines.org). All methods were performed in accordance with the relevant guidelines and regulations.

### Haematoxylin and eosin (H&E) and immunohistochemical (IHC) staining

Mouse tumour tissue was fixed in formalin, embedded in paraffin and cut into 4 μm sections. The sections were deparaffinized with xylene and hydrated in a descending ethanol series For H&E staining, the sections were stained with haematoxylin and eosin. For IHC staining, the sections were incubated with 0.3% H_2_O_2_ for 15 min to reduce endogenous peroxidase activity. Nonspecific binding was blocked with bovine serum albumin (BSA) for 30 min, and the sections were incubated with primary antibodies against Hp1α/β (1:200) or HMGB1 (1:200) at 4 °C overnight. Following incubation with a biotinylated secondary antibody for 30 min at 37 °C, antibody binding was visualized by 3, 3′-diaminobenzidine (DAB) staining.

### Flow cytometry analysis of mitochondrial membrane potential

The mitochondrial membrane potential was evaluated using JC-1 dye according to the manufacturer’s instructions. Briefly, cells were treated with Doc and diacerein, and then, 1 × 10^5^ cells were harvested and resuspended in 1 ml of DMEM/F12 medium. After incubation with JC-1 dye (2.5 mg/l) for 30 min under standard cell culture conditions, the fluorescence intensity of both mitochondrial JC-1 monomers and aggregates was detected by using a FACS Canto® Π flow cytometer with BD FACSDiva Software. The change in mitochondrial membrane potential in each group of cells was calculated based on the percentage of JC-1 monomers^18^.

### Flow cytometry analysis of DNA damage response signaling

After treatment with Doc and diacerein, DNA damage response signaling was assessed by detecting the expression of γ-H2A.X (Cell Signaling Technology) according to the manufacturer’s instructions. Briefly, cells were collected, fixed with 4% formaldehyde for 15 min and permeabilized with 2 ml ice-cold 90% methanol for 2 h at − 20 °C. After two washes with PBS to remove methanol, the cells were resuspended in 100 μl of PBS containing 0.5% BSA. The cells were incubated with γ-H2A.X (Alexa Fluor® 488 Conjugate) antibody for 1 h. After PBS washes to remove the excess antibody, the cells were resuspended in 300 µl of PBS and the expression of γ-H2A.X was analysed by a FACS Canto® Π flow cytometer with BD FACSDiva Software.

### RT‒qPCR analysis of mRNA levels

Total RNA was extracted from cells by RNAiso Plus (TaKaRa Bio, Japan) following the manufacturer’s instructions. A total of 1 µg RNA was used as template for a reverse transcription reaction using a reverse transcription kit (TaKaRa Bio). Generated cDNA was then analysed by qPCR using TB Green Premix Ex Taq (TaKaRa Bio) and quantified using ABI 7500 real-time PCR systems (Thermo Fisher Scientific) following the PCR thermocycling conditions: initial denaturation at 95 °C for 5 min; subsequent 40 cycles of 95 °C for 30 s (s) and 60 °C for 40 s. The mRNA expression of the target gene was analysed using the 2^−ΔΔCt^ method^19^ and determined by normalization to *GAPDH* (Homo) and *β-Actin* (Mus). The primers used are listed in the Supplementary material (Additional file 2 for Table S3).

### Statistical analysis

Each experiment was repeated at least three times, and all results of the bar graphs are represented as the mean ± standard deviation (S.D.). Statistical analysis was performed by SPSS software version 20.0. Student’s t test was used for comparisons between two groups, and comparisons among multiple groups were performed using one-way analysis of variance (ANOVA) followed by LSD post-hoc test. Values of *P* < 0.05 were considered statistically significant. In the graphed data *or^#^, **or^##^ and ***or^###^ denote values of *P* < 0.05, 0.01 and 0.001, respectively.

## Results

### Doc induced G2/M cell cycle arrest and the formation of PGCCs

**Figure 1:**
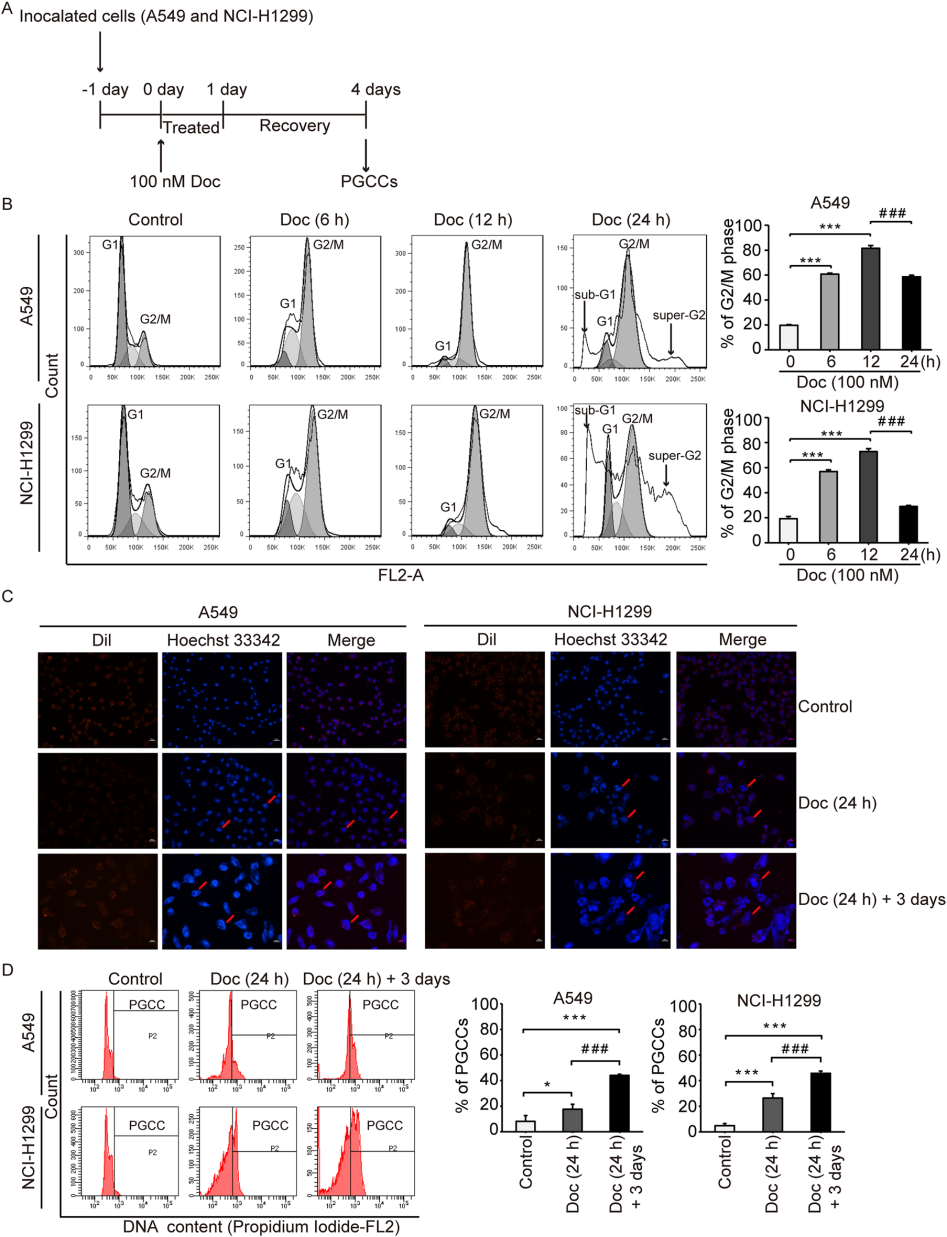
Doc induced G2/M cell cycle arrest and the formation of PGCCs. (**A**) Experimental design. The NSCLC cell lines A549 and H1299 cells were treated with Doc at 100 nM for 24 h, and then cultured in a drug-free medium for three days. (**B**) Cell cycle progression was detected by flow cytometry and analysed by Flow Jo 7.6 software after Doc treatment for 6 h, 12 h and 24 h. The percentage of cells in G2/M phase was presented in the histograms. (**C**) The morphology of cells was observed by a fluorescence microscope after staining the cells with Dil and Hochest 33,342 (× 200). Red arrows indicate the cells with giant nucleus. Bar = 100 μm. (**D**) DNA content of cells was analysed by flow cytometry and the region marked by black line (P2) shows the polyploid cells (DNA > 4N). Bar graphs showed the percentage of PGCCs (polyploid cells). N = 3, data was shown as mean ± SD. One-way ANOVA was used to determine statistical significance: *P < 0.05, ***P < 0.001 and ^###^*P* < 0.001.

The NSCLC cell lines A549 and NCI-H1299 were treated with Doc for 24 h and were allowed to recover for another three days. The experimental design is shown in Fig. 1A. To determine the influence of Doc on cell fate, we first analysed the cell cycle distribution. As shown in Fig. 1B, the fractions of A549 and NCI-H1299 cells in G2/M phase progressively increased from 19.73 ± 0.51 and 19.19 ± 1.76% at 0 h to 60.76 ± 0.83 and 56.90 ± 1.33% at 6 h (6 h vs. 0 h, *P* < 0.05) and 81.53 ± 2.25 and 72.82 ± 2.33% at 12 h (12 h vs. 0 h, *P* < 0.05). However, sub-G1 and super-G2 fractions were found after Doc exposure for 24 h (Fig. 1B and Supplementary, Additional file 3 for Fig. S1), so the fractions of A549 and NCI-H1299 cells in G2/M phase decreased significantly to 60.79 ± 8.20 and 31.05 ± 4.17% at 24 h (24 h vs. 12 h, *P* < 0.05). These data indicated that Doc induced G2/M cell cycle arrest in both cell lines followed by cell death and polyploidy.

Interestingly, the morphology of cells exposed to Doc for 24 h had greatly changed from spindle to flat, and the size of these cells became larger along with prolongation of the culture time (Supplementary, Additional file 4 for Fig. S2). Therefore, we further analysed the morphology of the Doc-induced cells by using Dil and Hoechst 33342 dye. The size and nuclei of the cells increased after Doc treatment and reached their largest sizes in the Doc (24 h) + 3 days group (Fig. 1C). Multiple nuclei or giant nuclei were observed even in a single enlarged cell, which was designated PGCC (Fig. 1C). The DNA content of A549 and NCI-H1299 cells treated with Doc was further analysed by flow cytometry. As shown in Fig. 1D, the percentage of PGCCs increased greatly from 8.20 ± 4.53 and 4.83 ± 1.72% in the control group to 17.60 ± 3.86 and 26.37 ± 3.51% in the Doc (24 h) group and to 44.07 ± 0.90 and 45.80 ± 1.73% in the Doc (24 h) + 3 days group, which was significantly more than that in the control group and Doc (24 h) group (*P* < 0.05). Collectively, these data suggested that PGCCs could be generated from NSCLC cells induced with Doc.

### Doc reduced the expression of key proteins related to proliferation

**Figure 2:**
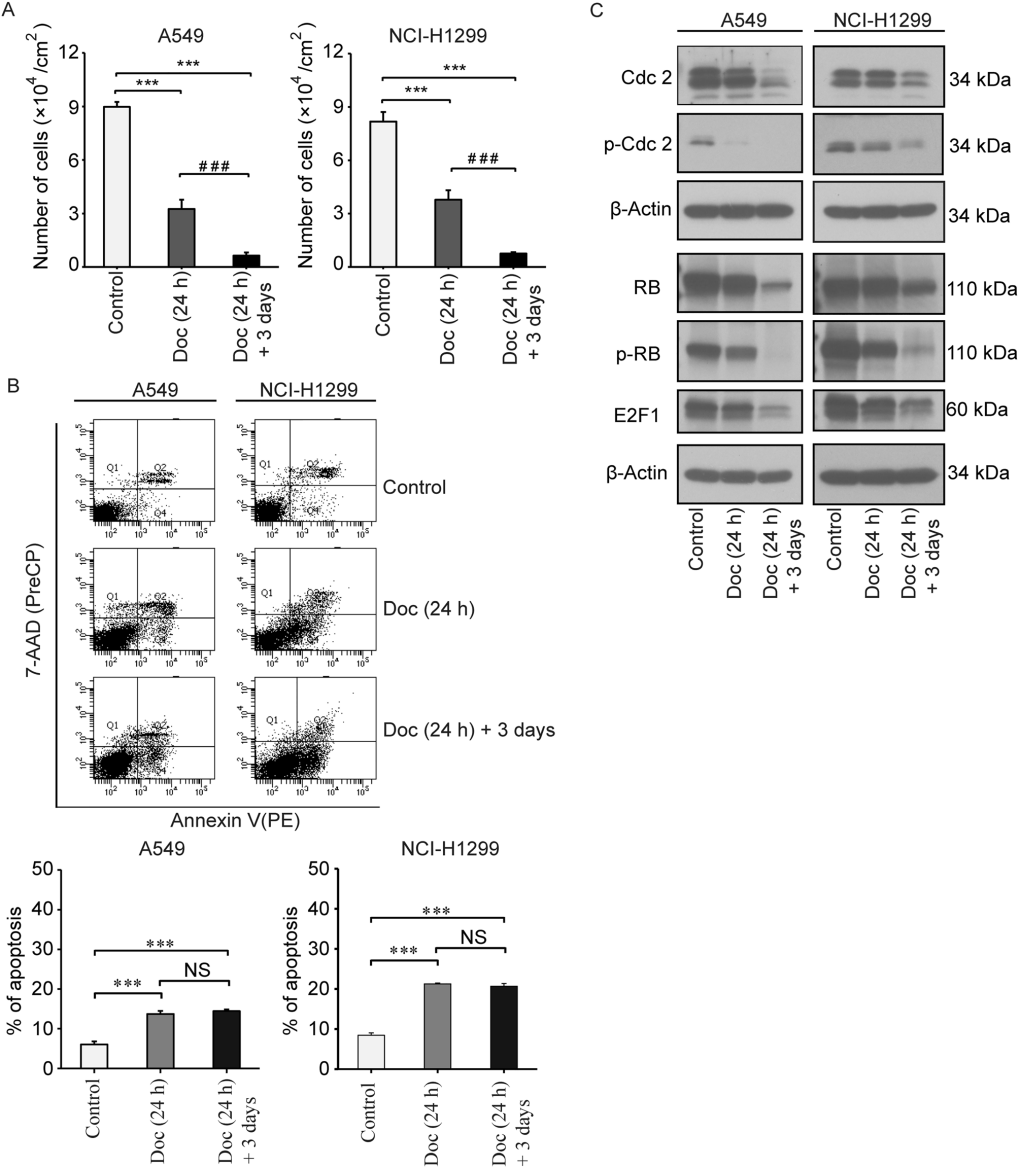
Doc reduced the expression of key proteins related to cell cycle progression and proliferation. (**A**) The number of cells was counted in a hemocytometer chamber under a microscope and presented in the histograms. (**B**) Cell viability was evaluated by flow cytometry with 7-AAD and PE Annexin V double staining. Q2: early apoptosis; Q4: late apoptosis; Q2 + Q4: apoptotic subgroup; Q3: viable subgroup. Bar graphs showed the percentage of apoptosis. (**C**) Proteins were extracted and the expression levels of Cdc2, p-Cdc2, RB, p-RB and E2F1 were then analysed by western blotting. β-Actin was used as loading control. N = 3, data was shown as mean ± SD. One-way ANOVA was used to determine statistical significance: ***P < 0.001 and ^###^*P* < 0.001.

Consistent with cell cycle arrest, Doc inhibited the proliferation of A549 and NCI-H1299 cells. As shown in Fig. 2A, the number of cells was reduced from 8.98 ± 0.28 and 8.18 ± 0.54 (× 10^4^ cells/cm^2^) in the control group to 3.24 ± 0.53 and 3.78 ± 0.54 (× 10^4^ cells/cm^2^) in the Doc (24 h) group and to 0.64 ± 0.17 and 0.75 ± 0.08 (× 10^4^ cells/cm^2^) in the Doc (24 h) + 3 days group, significantly more than that in the control group and Doc (24 h) group (*P* < 0.05). Moreover, the percentage of apoptosis in A549 and HCI-H1299 cells increased from 6.03 ± 0.76 and 8.40 ± 0.62% in the control group to 13.73 ± 0.75 and 21.27 ± 0.21% in the Doc (24 h) group (Fig. 2B). This result was consistent with the increase in sub-G1 and super-G2 fractions (Supplementary, Additional file 3 for Fig. S1). With the increase in DNA content and the formation of PGCCs after three days of recovery, the percentage of apoptosis in A549 and HCI-H1299 cells was not further increased (Figs. 1C, 2B). These results suggested that Doc inhibited the proliferation of NSCLC cells by inducing cell death and polyploidy.

Since Doc not only induced cell cycle arrest but also inhibited cell proliferation, we further investigated the effect of Doc on the expression of key proteins related to the cell cycle and proliferation. Cdc2 (CDK1) is a key regulator of the G2/M phase transition^20,21^, and the inhibition of CDK can block the phosphorylation of retinoblastoma protein (RB), thus preventing the transcription of proliferative genes mediated by E2F^22^. Therefore, the expression of Cdc2, p-Cdc2, RB, p-RB and E2F1 was analysed by western blotting. As shown in Fig. 2C, the protein levels of Cdc2 and p-Cdc2 were reduced after Doc treatment, particularly in the Doc (24 h) + 3 days group. Correspondingly, lower expression of RB and p-RB was observed in the Doc (24 h) + 3 days group (Fig. 2C). The protein level of E2F1 was also reduced, particularly in the Doc (24 h) + 3 days group (Fig. 2C). Thus, Doc could induce NSCLC cells to form PGCCs by modulating the expression of key regulatory proteins related to the cell cycle and proliferation.

### PGCCs induced by Doc experienced senescence

**Figure 3:**
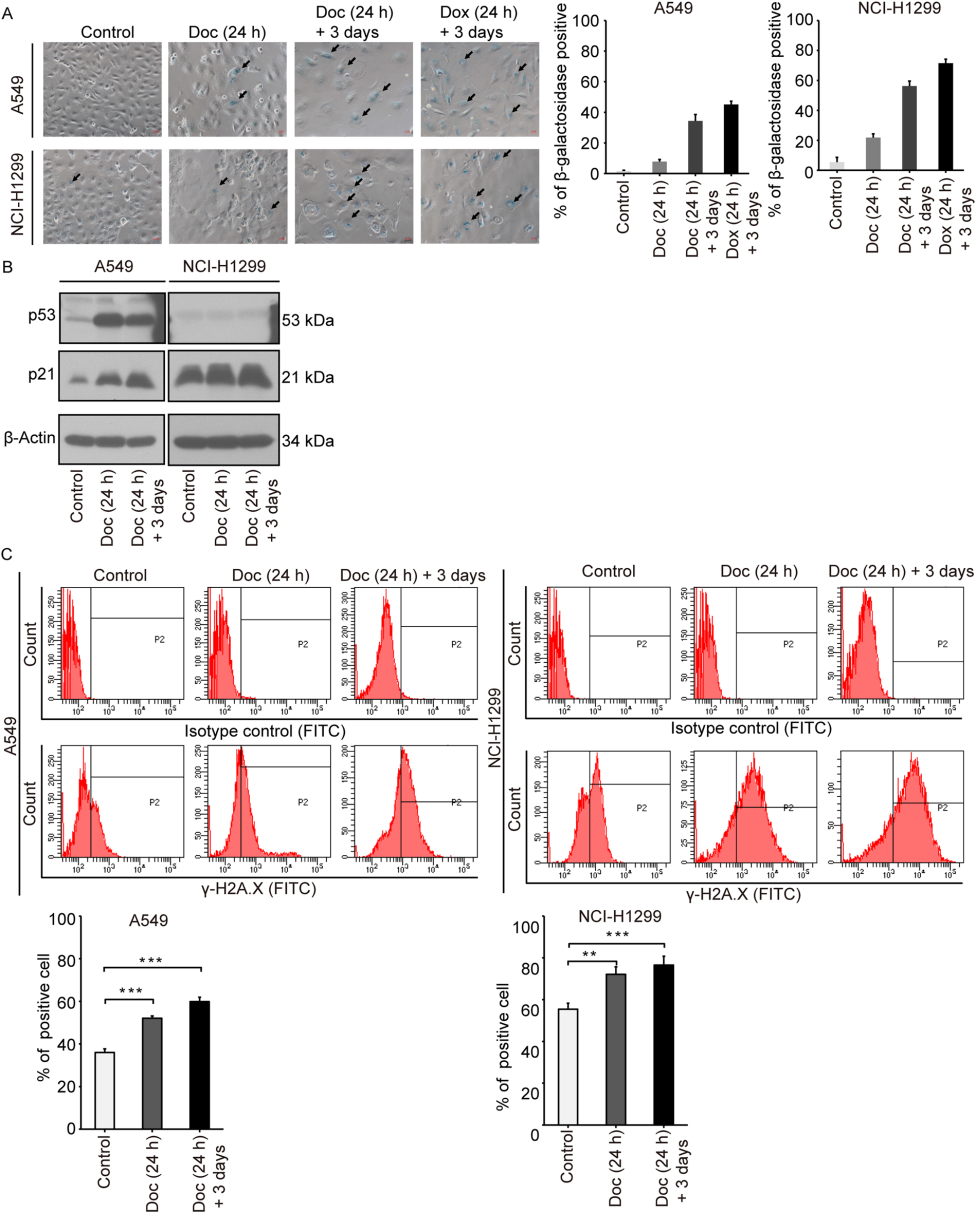
PGCCs induced by Doc experienced senescence. (**A**) Cells were stained with a senescence-associated β-galactoside (SA-β-Gal) staining kit, and images were acquired by an inverted microscope (× 200). Black arrows indicate the SA-β-Gal-positive cell. Bar = 100 μm. Doxorubicin (132 nM) was used as a positive control to display senescence induction. (**B**) Proteins were extracted and the protein level of p53 and p21 was then analysed by western blotting. β-Actin was used as loading control. (**C**) The expression of γ-H2A.X was analysed by flow cytometry and the region marked by black line (P2) showed the γ-H2A.X-positive cells. Bar graphs showed the percentage of γ-H2A.X-positive cells. N = 3, data was shown as mean ± SD. One-way ANOVA was used to determine statistical significance: **P < 0.01, ***P < 0.001 and ^###^*P* < 0.001.

Doc-induced PGCCs revealed some characteristic features of senescence, such as enlarged and flattened morphology (Supplementary, Additional file 4 for Fig. S2), as well as irreversible cell cycle arrest and proliferation inhibition (Figs. 1B, 2A). To confirm that Doc-induced PGCCs underwent senescence, we used an SA-β-Gal staining kit to detect the activity of β-galactosidase. Doxorubicin (132 nM) was included as a positive control to display senescence induction^23^. As shown in Fig. 3A, A549 and NCI-H1299 cells became enlarged and flattened after Doc treatment and showed increased β-galactosidase activity (*P* < 0.05). Positive staining of β-galactosidase was mainly concentrated in large cells, particularly in the Doc (24 h) + 3 days group (Fig. 3A). Therefore, Doc induced a senescent phenotype with the development of PGCCs.

The p53/p21 signaling pathway plays an important role in the regulation of cell cycle progression, and activated p53/p21 signaling is typical of cell stress/senescence^24,25^. Thus, the expression of the p53 and p21 proteins was assessed by western blotting. Following treatment with Doc, the protein level of p21 was significantly increased, particularly in the Doc (24 h) + 3 days group (Fig. 3B). Although Doc increased the protein level of p53 in A549 cells, the induction of p21 was p53-independent since NCI-H1299 cells were p53 null (Fig. 3B). These data suggested that the expression of p21 might be related to the formation of PGCCs and the occurrence of senescence. The DNA damage response is a major mechanism that elicits senescence^24^. Therefore, the expression of γ-H2A.X (a marker of DNA damage response) was analysed by flow cytometry. As shown in Fig. 3C, the percentage of γ-H2A.X-positive cells in A549 and NCI-H1299 cells was increased from 36.00 ± 1.65 and 55.43 ± 2.84% in the control group to 52.07 ± 1.03 and 72.13 ± 3.52% in the Doc (24 h) group and further to 59.90 ± 2.04 and 76.57 ± 4.25% in the Doc (24 h) + 3 days group, significantly more than that in the control group (*P* < 0.05). These data suggested that the activation of the DNA damage response might be related to the formation of PGCCs and the occurrence of senescence. Taken together, these data clearly indicated that Doc not only induced PGCCs but also promoted senescence, and these two processes might be controlled by p21 and the DNA damage response signal.

### Doc promoted the expression of IL-1β in vitro and in vivo

**Figure 4:**
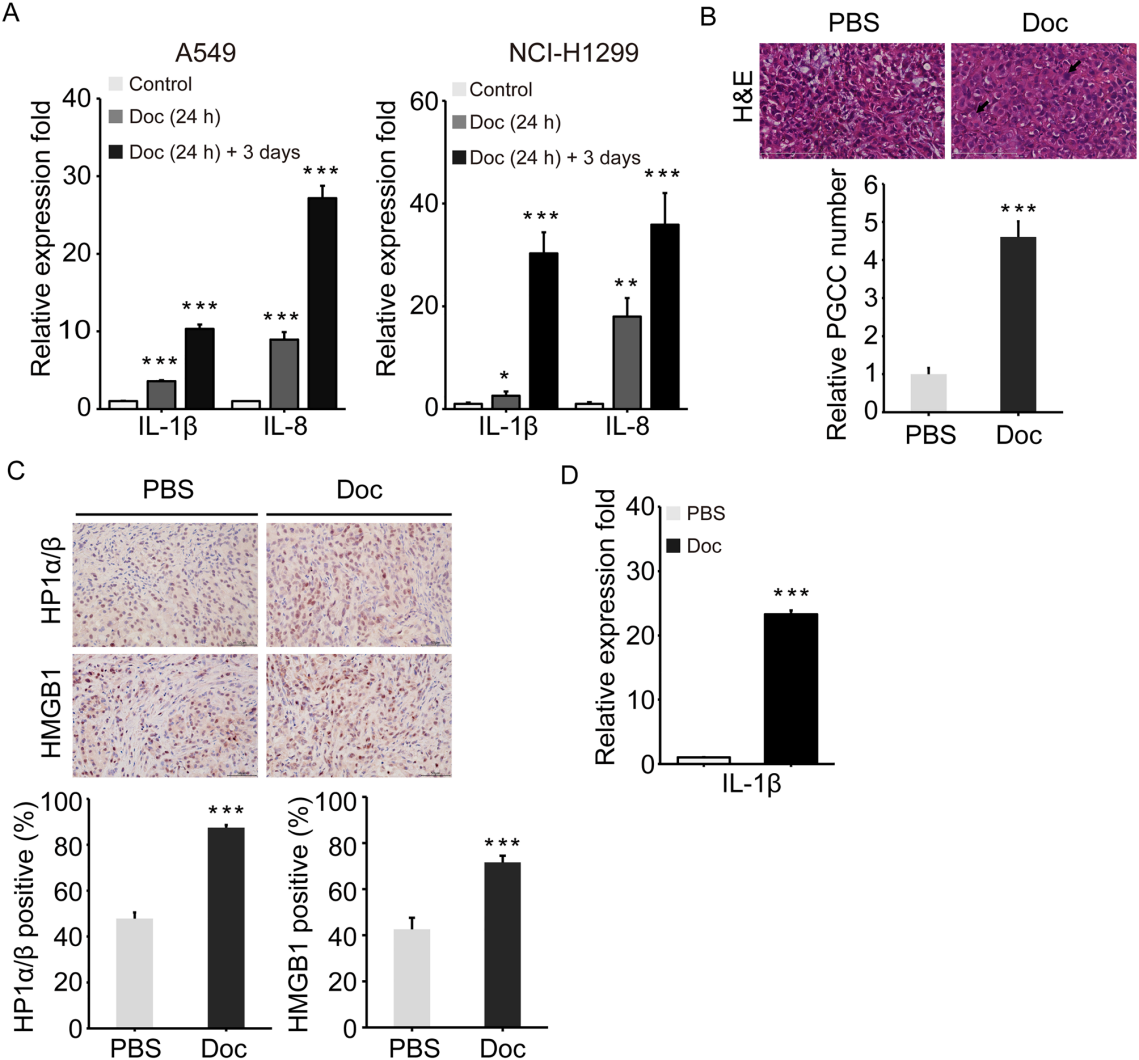
Doc promoted the expression of IL-1β in vitro and vivo. (**A**) The mRNA level of *IL-1β* and *IL-8* was determined by RT‒qPCR. The values were normalized to *GADPH* (Homo sapiens) and related to the control group. (**B**) Histology features after H&E staining from the A549 cells xenografted nude mice with or without Doc treatment. Bar = 100 μm. Bar graphs showed the relative PGCC number. The percentage of PGCC (approximately three times the average mononuclear area) number was quantified and normalized to the PBS (control) group (N = 15). (**C**) Immunohistochemistry analysis of HP1α/β and HMGB1 expressed in the A549 cells xenografted nude mice tumour tissues. Bar = 50 μm. Bar graphs showed the percentage of positive cells. The expression of HP1α/β and HMGB1 was quantified according to the percentage of positive cells (N = 8). (**D**) Relative mRNA expression of *IL-1β* in the A549 cells xenografted nude mice tumour tissues was determined by RT‒qPCR. The values were normalized to *Actin* (Mus musculus) and relative to the PBS (control) group. N = 3, data was shown as mean ± SD. One-way ANOVA and student’s t test were used to determine statistical significance: *P < 0.05, **P < 0.01 and ***P < 0.001.

Cellular senescence is accompanied by a striking increase in the secreted levels of soluble factors^13^. We were particularly interested in exploring the contribution of senescence-associated proinflammatory cytokines to the formation of PGCCs and senescence. Therefore, the levels of *IL-1β, IL-6* and *IL-8* mRNA were analysed by RT‒qPCR, since these important inflammatory molecules are involved in senescence and are predominantly expressed in PGCCs^26^. As expected, *IL-1β* mRNA levels in A549 and NCI-H1299 cells were increased by 3.6 ± 0.1- and 2.6 ± 0.8-fold in the Doc (24 h) group and 10.3 ± 0.5- and 30.3 ± 4.1-fold in the Doc (24 h) + 3 days group compared to that of the control group (Fig. 4A, *P*  < 0.05). Moreover, *IL-8* mRNA levels in A549 and NCI-H1299 cells were increased by 8.9 ± 1.0- and 18.0 ± 3.6-fold in the Doc (24 h) group and 27.2 ± 1.6- and 35.9 ± 6.2-fold in the Doc (24 h) + 3 days group compared to that of the control group (Fig. 4A, *P* < 0.05). However, the *IL-6* mRNA level was not significantly changed by Doc treatment (data not shown). These data indicated that Doc treatment increased the expression of IL-1β and IL-8 with the development of PGCCs in vitro.

The A549 xenograft tumour model was established to investigate the effect of Doc on tumour cells in vivo. H&E staining showed that Doc treatment led to the formation of PGCCs with bizarre nuclei (Fig. 4B). Furthermore, Doc markedly promoted the expression of HP1α/β and HMGB1 (marker proteins of senescence) at the histological level (Fig. 4C). These data suggested that Doc induced the formation of PGCCs and senescence in vivo. Based on the data observed in vitro, we further analysed the effect of Doc on the expression of *IL-1β* and *IL-8 *in vivo. Since IL-8 is deleted in mice, *IL-1β* mRNA level was analysed in tumour-bearing mice treated with Doc by RT‒qPCR. Consistent with the in vitro results, *IL-1β* mRNA level was significantly increased by 23.3 ± 0.6-fold after Doc treatment compared with that of the PBS group (Fig. 4D, *P* < 0.05). Taken together, these data suggested that Doc promoted senescence and the expression of IL-1β with the development of PGCCs in vivo.

### Inhibition of IL-1β prevented senescence and facilitated the development of PGCCs

**Figure 5:**
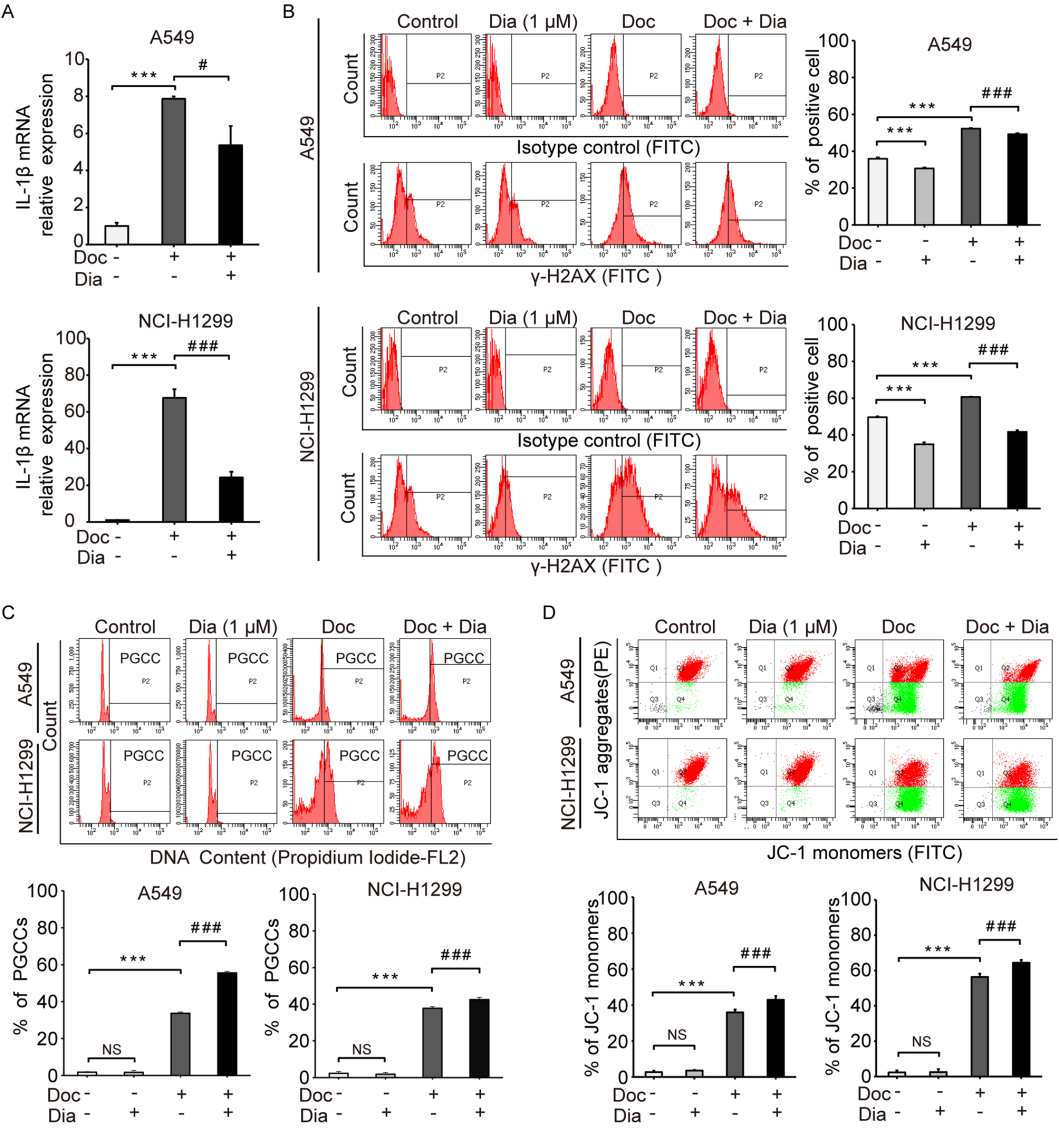
Inhibition of IL-1β hindered senescence and facilitated the development of PGCCs. (**A**) Relative mRNA expression of *IL-1β* was determined by RT–qPCR. The values were normalized to *GADPH* and relative to the control group. (**B**) The expression of γ-H2A.X was analysed by flow cytometry and the region marked by black line (P2) showed γ-H2A.X-positive cells. Bar graphs showed the percentage of positive cells. (**C**) DNA content of cells was analysed by flow cytometry and the region marked by black line (P2) shows the polyploid cells (DNA > 4N). Bar graphs showed the percentage of PGCCs (polyploid cells). (**D**) Mitochondrial membrane potential was determined by flow cytometry. Bar graphs showed the percentage of JC-1 monomers. N = 3, data was shown as mean ± SD. One-way ANOVA was used to determine statistical significance: ***P < 0.001, ^#^*P* < 0.05 and ^###^*P* < 0.001.

To investigate the effect of IL-1β on the formation and development of PGCCs, we used diacerein as an inhibitor of IL-1β. As shown in Fig. 5A, diacerein partially blocked the induction of *IL-1β* by Doc. The effect of inhibiting IL-1β on cell senescence was assessed by analysing the expression of γ-H2A.X. Diacerein significantly reduced the expression of γ-H2A.X, whether alone or in combination with Doc. As shown in Fig. 5B, treatment with diacerein alone reduced the percentage of γ-H2A.X-positive cells in A549 and NCI-H1299 cells from 35.87 ± 0.76 and 49.70 ± 0.46% in the control group to 30.70 ± 0.53 and 34.93 ± 1.12% in Dia group (Dia group vs. control group, *P* < 0.05). Importantly, treatment with Doc and diacerein in combination reduced the percentage of γ-H2A.X-positive cells from 52.23 ± 0.31 and 60.63 ± 0.21% in the Doc group to 49.23 ± 0.55 and 41.70 ± 0.95% in the Doc + Dia group (Doc + Dia group vs. Dia group, *P* < 0.05). The effect of IL-1β inhibition on the development of PGCCs was further evaluated by analysis of DNA content with flow cytometry. As shown in Fig. 5C, treatment with diacerein alone did not increase the percentage of PGCCs in A549 and NCI-H1299 cells (Dia group vs. control group, *P* > 0.05). However, treatment with Doc and diacerein in combination increased the percentage of PGCCs in A549 and NCI-H1299 cells from 33.70 ± 0.70 and 37.77 ± 0.93% in the Doc group to 55.53 ± 0.81 and 42.53 ± 1.25% in the Doc + Dia group (Doc + Dia group vs. Doc group, *P* < 0.05). These data indicated that IL-1β inhibition synergistically promoted the formation of PGCCs with the induction of Doc.

The mitochondrial membrane potential was measured to assess the effect of IL-1β on cell viability by using JC-1 dye. As shown in Fig. 5D, JC-1 dye existed in the form of aggregates in the control group and Dia group of A549 and NCI-H1299 cells (Dia group vs. control group,* P* > 0.05), suggesting that treatment with diacerein alone had no effect on cell viability. After treatment with Doc alone, the proportions of the JC-1 monomeric form in A549 and NCI-H1299 cells were significantly increased from 2.70 ± 0.75 and 2.37 ± 1.17% in the control group to 35.97 ± 1.62 and 56.40 ± 1.78% in the Doc group (Fig. 5D, Doc group vs. control group,* P* < 0.05). Notably, treatment with Doc and diacerein in combination further increased the proportions of the JC-1 monomeric form in A549 and NCI-H1299 cells to 42.97 ± 2.08 and 64.40 ± 1.49%, significantly more than that in the Doc group (Fig. 5D, Doc + Dia group vs. Doc group, *P* < 0.05). These data suggested that Doc caused depolarization of the mitochondrial transmembrane potential to induce apoptosis and that IL-1β inhibition synergistically enhanced the proapoptotic effect of Doc.

## Discussion

Chemotherapy is one of the traditional therapeutic methods for malignant tumour. Although chemotherapy drugs can eliminate cancer cells by inducing cell death via apoptosis, autophagy or mitotic catastrophe, drug resistance often and inevitably occurs^27^. Chemotherapy drugs, such as Doc, can damage the mitotic spindle and shut down mitosis, which leads to mitotic catastrophe and substantial cell death^26^. However, chemotherapy drugs also lead to a switch from the mitotic cell cycle to the endoreplication cell cycle, which leads to the formation of PGCCs, a more conducive form to adjust to the harsh living environment^28^. The formation of PGCCs after therapeutic intervention with chemotherapy drugs, including Doc, has been well described. These PGCCs can acquire a proinflammatory secretory phenotype and contribute to the acquisition of chemoresistance^29^. Here, we found that Doc could induce G2/M cell cycle arrest and cell death in A549 and NCI-H1299 cells. Concurrently, Doc induced PGCCs, which showed typical features of senescence. The inflammatory cytokine IL-1β in the SASP was increased significantly with the development of PGCCs and contributed to Doc resistance.

Cellular senescence was originally identified as a stable state of cell cycle arrest and proliferation inhibition, which is also considered a stress response that can be triggered in cancer cells in response to irradiation or chemotherapy drugs^30,31^, termed therapy-induced senescence (TIS). Cellular senescence has long been regarded as synergistic with the prevention and control of malignant tumour and is increasingly recognized as an important concept in cancer biology^32^. However, TIS does not aprrear to be beneficial, as it may lead to chemoresistance and cancer recurrence. Jackson et al. found that senescence could impair the efficiency of chemotherapy and promote the recurrence of breast cancer^33^. Wang et al. demonstrated that a marker of TIS in vivo following neoadjuvant therapy predicted adverse clinical outcomes in patients with locally advanced NSCLC^27^. Recent studies have revealed that selectively targeting and effectively eliminating senescent cells in vivo can significantly promote therapeutic outcomes and elongate the lifespan of experimental animals^34,35^. In clinical practice, senescence may be a key point to examine after chemotherapy. In this study, we focused on the effect of Doc on cellular senescence. Our study suggests that Doc treatment leads to cell senescence and that the inflammatory cytokine IL-1β in the SASP contributes to Doc resistance. IL-1β is not only an important inflammatory factor secreted by senescent cells, but is also directly or indirectly involved in senescence. Ashraf et al. stressed that IL-1β is the most critical gene responsible for the direct induction of senescence to change the phenotypic and molecular characteristics of chondrocytes^36^. Li et al. and Chen et al. also demonstrated that IL-1β markedly increased the expression of SA-β-Gal^37,38^. In addition, IL-1β is central to the inflammatory response and activates a large number of signal pathways, including p38 MAPK, JNK, ERK and NF-κB. These signaling pathways can lead to the transcription of target genes involved in senescence, such as IL-6 and IL-8, which induce a self- and cross-reinforced senescence/inflammatory milieu^39^.

IL-1β is an important proinflammatory cytokine that is involved in stress and chronic inflammation^40^. Chronic inflammation is an important factor in carcinogenesis and tumour progression, and cancer-related inflammation is considered an important marker of cancer^41,42^. Although IL-1β has been extensively studied in immune cells, recent studies have shown that tumour cells also express IL-1β, which can be directly produced by cancer cells treated with a number of chemotherapeutic drugs^43^. However, IL-1β has been shown to have a positive effect on chemoresistance. Several studies have proposed a role for IL-1β in the poor responses to chemotherapy with consequent treatment failures^44,45^. Lin Lu et al. showed that IL-1β promotes the drug resistance of head and neck squamous cell carcinoma and melanoma cells^46^. Alexander et al. reported that chemoresistant cancer cells can release IL-1β, which maintains an NF-κB amplification loop responsible for chemoresistance^47^. Ji-Won Kim et al. found that high IL-1β levels are associated with shorter overall and progression-free survival for NSCLC patients treated with platinum-based combination chemotherapy^48^. IL-1β may promote drug resistance and tumour survival and is emerging as a prognostic factor for patients in response to targeted therapy, making IL-1β a possible target that may need to be taken into consideration.

In this study, we demonstrated for the first time that IL-1β plays an important role in the resistance of NSCLC to Doc chemotherapy via its involvement in the formation of PGCCs and the occurrence of senescence. Therefore, it is reasonable that targeting IL-1β in PGCCs may be a novel approach to overcome drug resistance.

### Supplementary Information

Supplementary Information 1.

Supplementary Information 2.

Supplementary Figure 1.

Supplementary Figure 2.

Supplementary Information 5.

Supplementary Information 6.

Supplementary Information 7.

## Data Availability

The datasets used and analysed during the current study are available from the corresponding author on reasonable request.
