# Supplementary material for: IL-1β is involved in docetaxel chemoresistance by regulating the formation of polyploid giant cancer cells in non-small cell lung cancer
Source: Sci Rep. 2023 Aug 7;13:12763. doi: 10.1038/s41598-023-39880-2 (PMC10406903; doi:10.1038/s41598-023-39880-2)
Supplement: Supplementary file 1 — Supplementary Information 1. [file 41598_2023_39880_MOESM1_ESM.doc]

**Additional file 1: Table S1 and S2. Reagents and antibodies**

Table S1 Reagents

| **Name** | **Manufacturer** | **Code Number** | **Dissolvant** | **Concentration** |
| --- | --- | --- | --- | --- |
| Docetaxel | Selleckchem | S1148 | DMSO | 50 mM |
| Diacerein | Selleckchem | S4267 | DMSO | 100 mM |
| Propidium iodide | Sigma Aldrich | P4107 | PBS | 10 mg/ml |
| JC-1 | Solarbio | J8030 | DMEM/F12 | 1 mg/ml |
| Doxorubicin | Selleckchem | E2516 | DMSO | 2 mg/ml |

Selleckchem: Radnor, PA, USA; Sigma Aldrich: St. Louis, MO, USA; Solarbio: Beijing, China.

DMSO: Dimethyl sulfoxide; PBS: Phosphate buffered saline; DMEM/F12: Dulbecco's Modified Eagle Medium/Nutrient Mixture F-12

Table S2 Antibodies

| **The primary antibody** | **Manufacturer** | **Code**  **Number** | **Dilution**  **ratio** | **Application** |
| --- | --- | --- | --- | --- |
| β-Actin | Santa Cruz Biotechnology | SC-47778 | 1:5000 | WB |
| E2F1 | Santa Cruz Biotechnology | SC-193 | 1:500 | WB |
| p53 | Cell Signaling Technology | 9282 | 1:500 | WB |
| p21 | Cell Signaling Technology | 2947 | 1:500 | WB |
| Cdc2 | Cell Signaling Technology | 9112 | 1:1000 | WB |
| p-Cdc2 | Cell Signaling Technology | 4539 | 1:500 | WB |
| RB | Cell Signaling Technology | 9309 | 1:300 | WB |
| p-RB | Cell Signaling Technology | 9308 | 1:300 | WB |
| Biotinylated Protein Ladder | Cell Signaling Technology | 7727 | No | WB |
| HP1α/β | Cell Signaling Technology | 2623 | 1:200 | IHC |
| HMGB1 | Cell Signaling Technology | 6893 | 1:200 | IHC |
| Phospho-Histone H2A.X (Ser139) | Cell Signaling Technology | 9719 | 1:50 | F |
| **The secondary antibody** | **Manufacturer** | **Code**  **Number** | **Dilution**  **ratio** | **Application** |
| Anti-rabbit IgG | Cell Signaling Technology | 7074 | 1:5000 | WB |
| Anti-mouse IgG | Cell Signaling Technology | 7076 | 1:5000 | WB |
| Anti-biotin | Cell Signaling Technology | 7075 | 1:2000 | WB |
| Goat anti-Rabbit IgG | Thermofisher | 31460 | 1:5000 | IHC |

Santa Cruz Biotechnology: San Jose, CA, USA; Cell Signaling Technology: Danvers, MA, USA; Thermofisher: Waltham, Ma, USA.

WB: West bolt; IHC: Immunohistochemistry; F: Flow cytometry
