# Supplementary material for: IL-1β is involved in docetaxel chemoresistance by regulating the formation of polyploid giant cancer cells in non-small cell lung cancer
Source: Sci Rep. 2023 Aug 7;13:12763. doi: 10.1038/s41598-023-39880-2 (PMC10406903; doi:10.1038/s41598-023-39880-2)
Supplement: Supplementary file 2 — Supplementary Information 2. [file 41598_2023_39880_MOESM2_ESM.doc]

**Additional file 2: Table S3. Primer sequence**

| **Name** | **F-Sequence** | **R- Sequence** |
| --- | --- | --- |
| **Homo sapiens** | | |
| **GAPDH** | CAACTTTGGTATCGTGGAAGGACT | TGATGTTCTGGAGAGCCCCG |
| **IL-1β** | TGAGCTCGCCAGTGAAATGAT | TGCTGTAGTGGTGGTCGGAG |
| **IL6** | TGCAATAACCACCCCTGACC | ATTTGCCGAAGAGCCCTCAG |
| **IL8** | ACATACTCCAAACCTTTCCACC | CCCTCTTCAAAAACTTCTCCAC |
| **Mus musculus** | | |
| **β-actin** | CCCTGAACCCCAAAGCTAACC | CACCGTCCCCAGAATCCAA |
| **IL-1β** | TGAAATGCCACCTTTTGACAGT | TGTGCTGCTGCGAGATTTGA |
| **IL6** | AACCACGGCCTTCCCTACTT | TTCTCATTTCCACGATTTCCC |
