# Supplementary material for: IL-1β is involved in docetaxel chemoresistance by regulating the formation of polyploid giant cancer cells in non-small cell lung cancer
Source: Sci Rep. 2023 Aug 7;13:12763. doi: 10.1038/s41598-023-39880-2 (PMC10406903; doi:10.1038/s41598-023-39880-2)
Supplement: Supplementary file 3 — Supplementary Figure 1. [file 41598_2023_39880_MOESM3_ESM.doc]

**
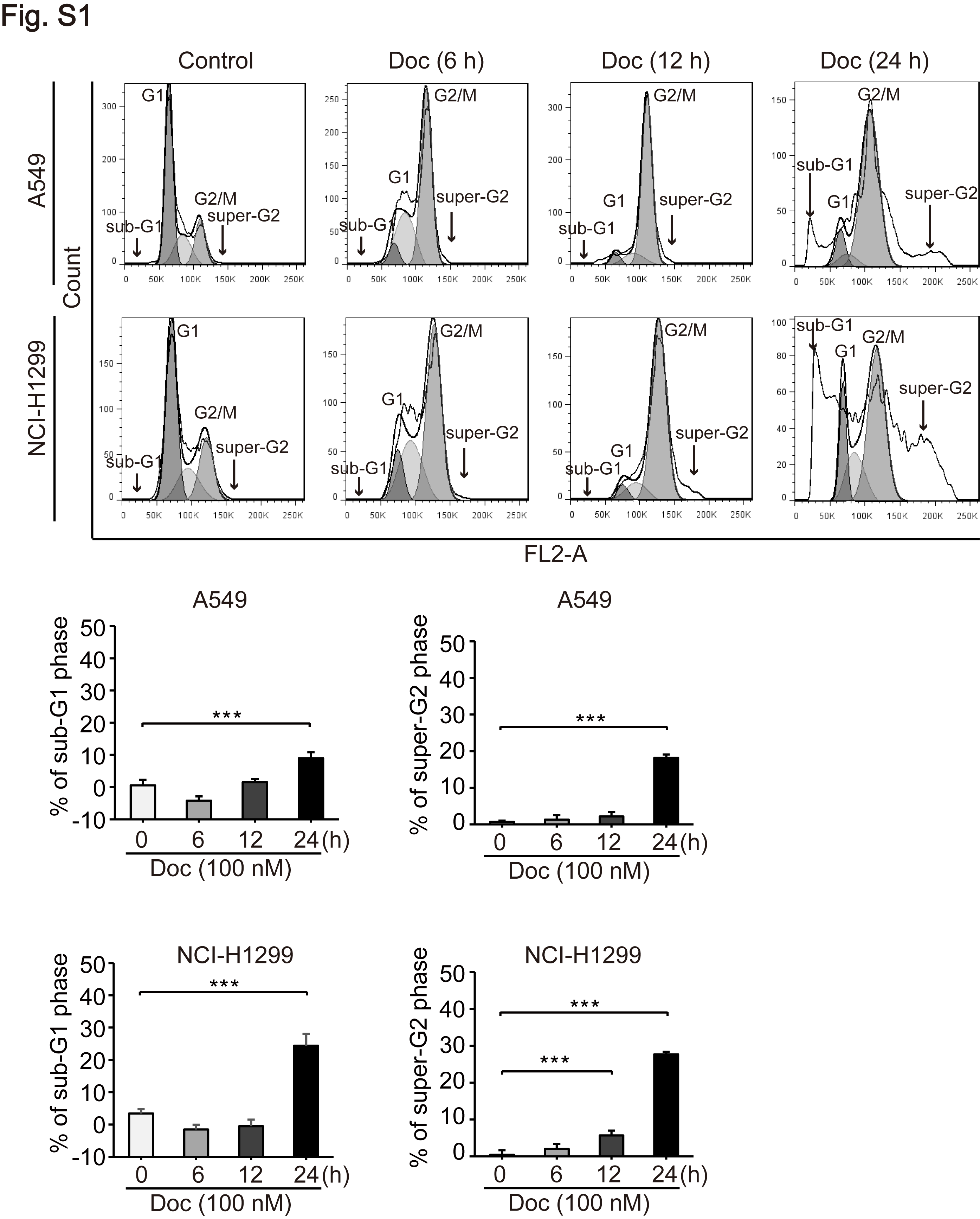
**

**Supplementary Figure 1. The effect of Doc on cell cycle distribution.**

The NSCLC cell lines A549 and H1299 were treated with Doc at 100 nM for 6 h, 12 h and 24 h. Cell cycle progression was detected by flow cytometry and analyzed by Flow Jo 7.6 software. The percentage of cells in sub-G1 and super-G2 phases was presented in the histograms. N=3, data was shown as mean ± SD. One-way ANOVA was used to determine statistical significance: ***P<0.001.
