# Supplementary material for: IL-1β is involved in docetaxel chemoresistance by regulating the formation of polyploid giant cancer cells in non-small cell lung cancer
Source: Sci Rep. 2023 Aug 7;13:12763. doi: 10.1038/s41598-023-39880-2 (PMC10406903; doi:10.1038/s41598-023-39880-2)
Supplement: Supplementary file 4 — Supplementary Figure 2. [file 41598_2023_39880_MOESM4_ESM.doc]

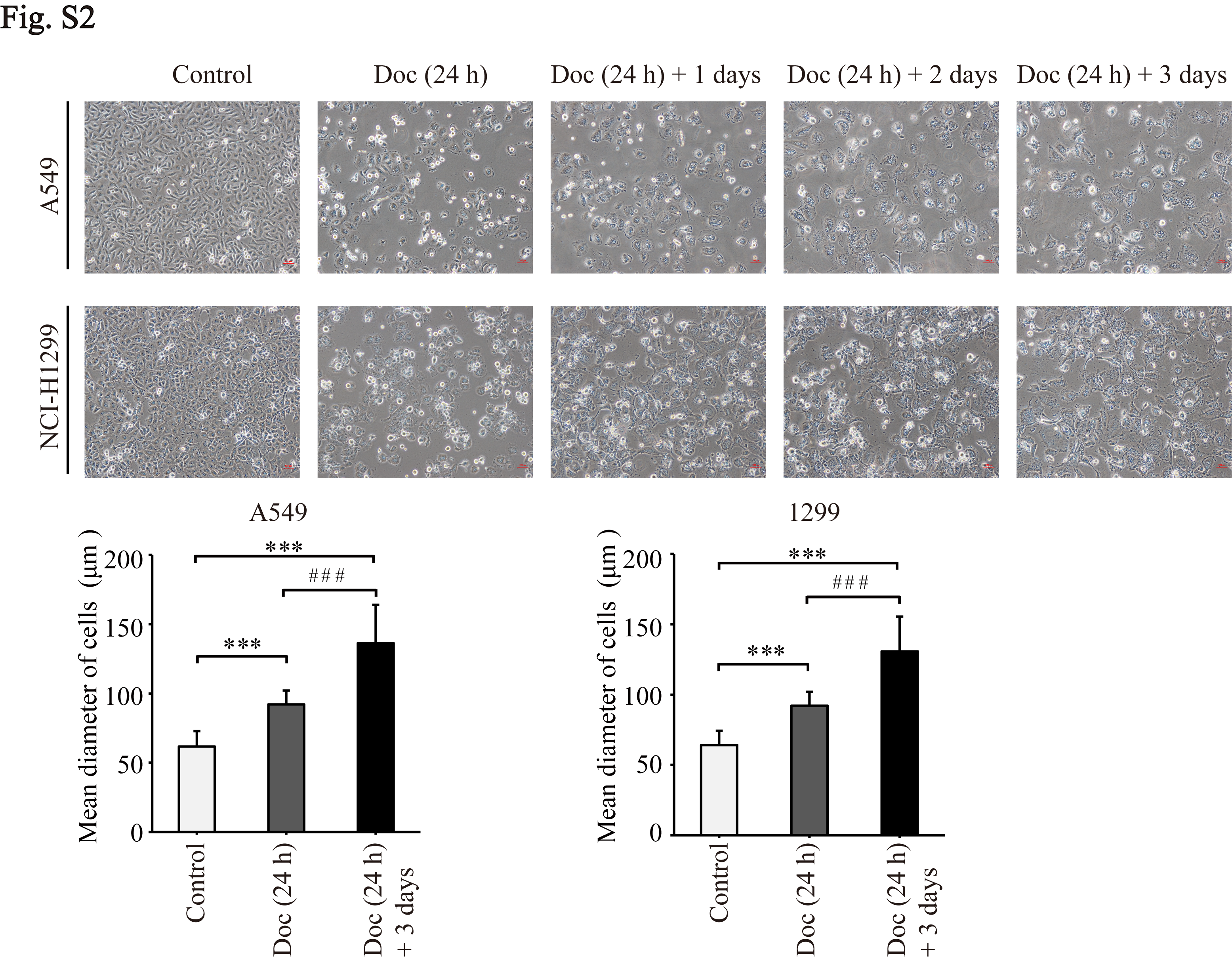


**Supplementary Figure 2. Doc increased the diameter of cells.**

A549 and NCI-H1299 cells were treated with Doc at 100 nM for 24 h and then recovered in regular medium for 3 days. The morphology of cells was observed by conventional light microscopy (×100). Bar = 100 μm. The diameter of cells was analyzed by image pro plus6.0 software. N=50, data were expressed as the mean ± SD. One-way ANOVA was used to determine statistical significance: ***P<0.001 vs. control group; ^###^P<0.001 vs. Doc (24 h) group.
