# Supplementary figures and images for: IL-1β is involved in docetaxel chemoresistance by regulating the formation of polyploid giant cancer cells in non-small cell lung cancer
Source: Sci Rep. 2023 Aug 7;13:12763. doi: 10.1038/s41598-023-39880-2 (PMC10406903; doi:10.1038/s41598-023-39880-2)

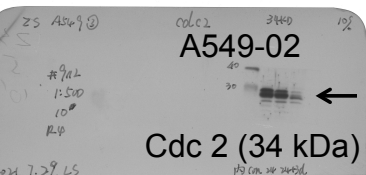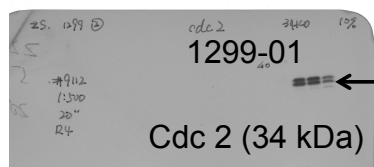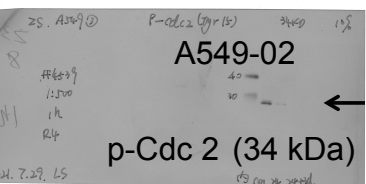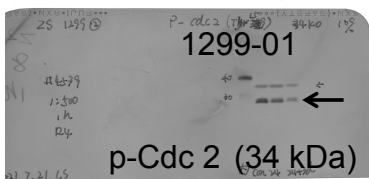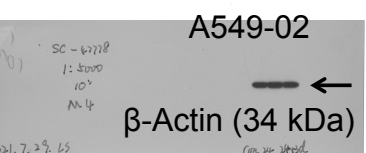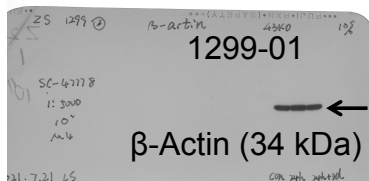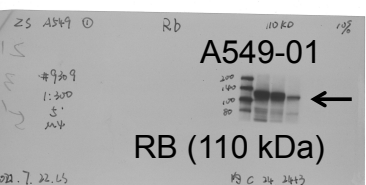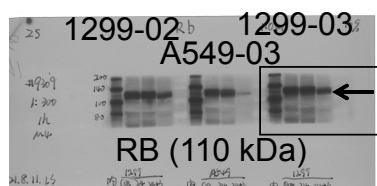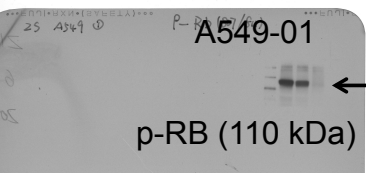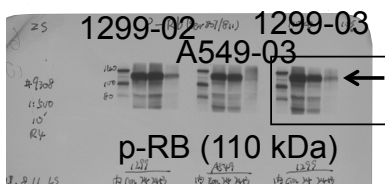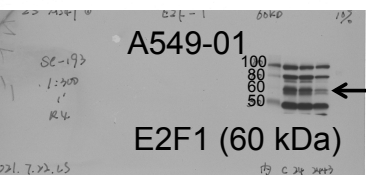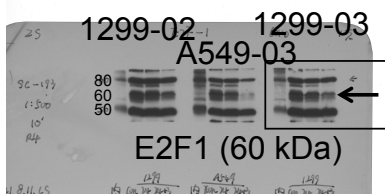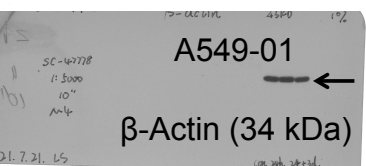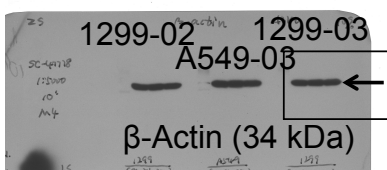

Supplement: Supplementary file 5 — Supplementary Information 5. [file 41598_2023_39880_MOESM5_ESM.pdf]

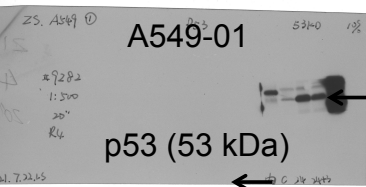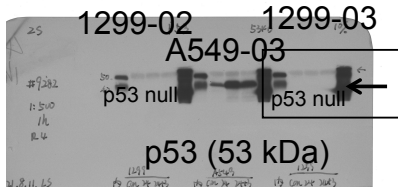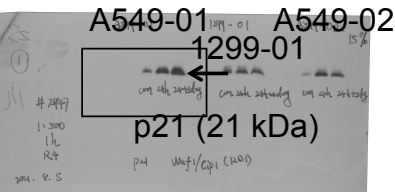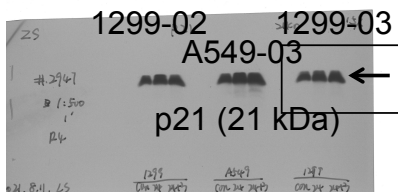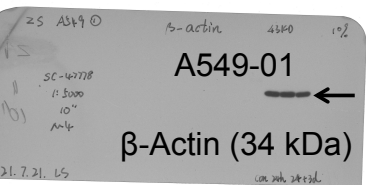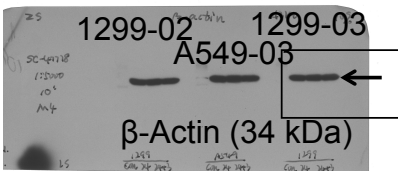

Supplement: Supplementary file 6 — Supplementary Information 6. [file 41598_2023_39880_MOESM6_ESM.pdf]

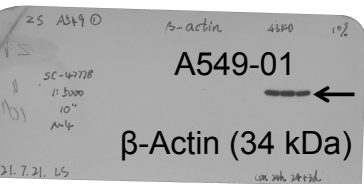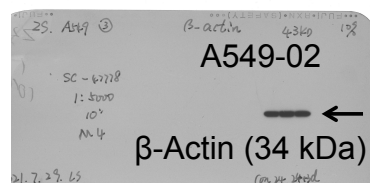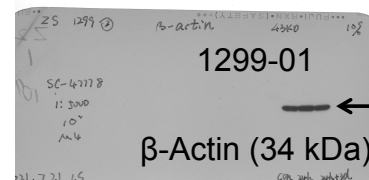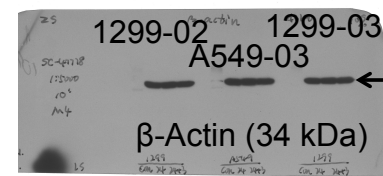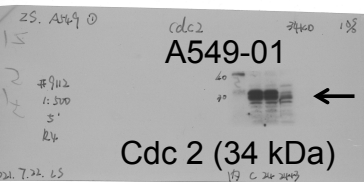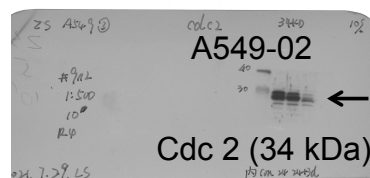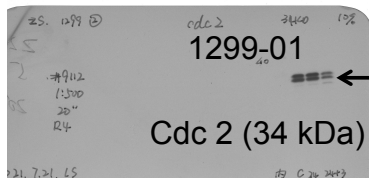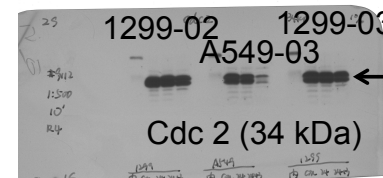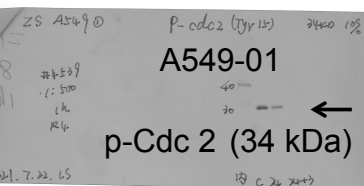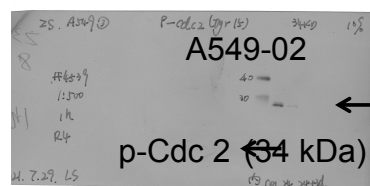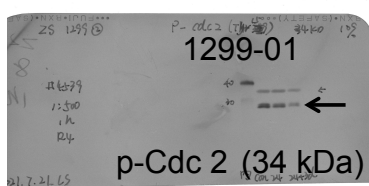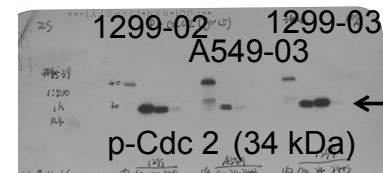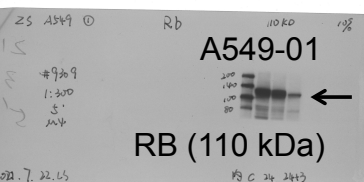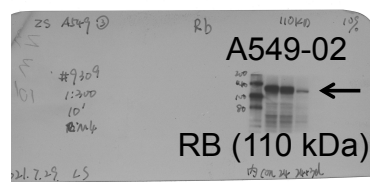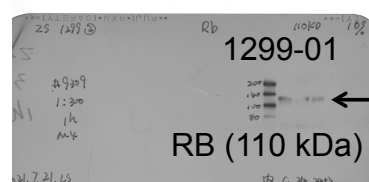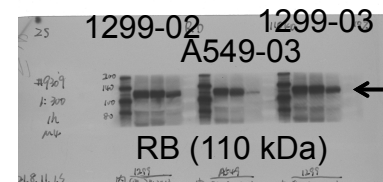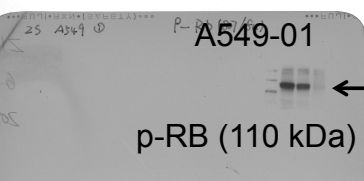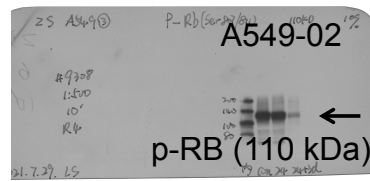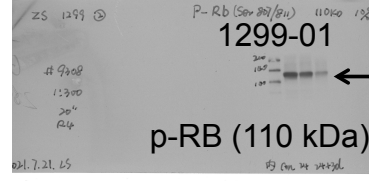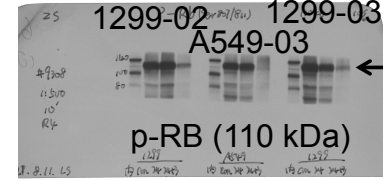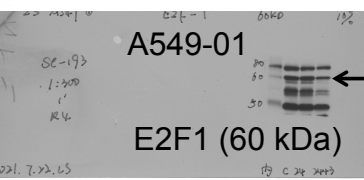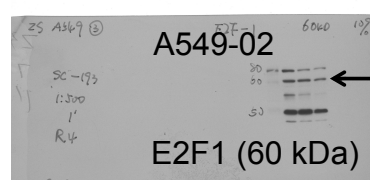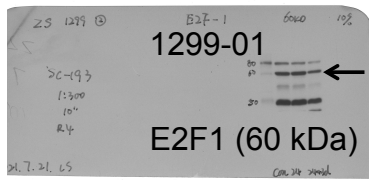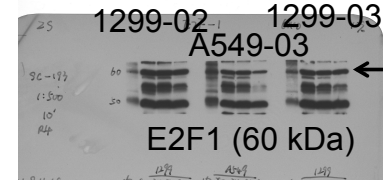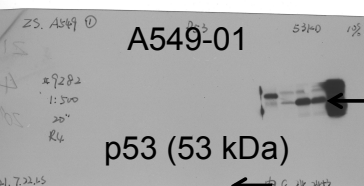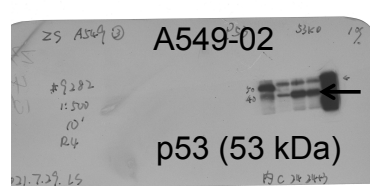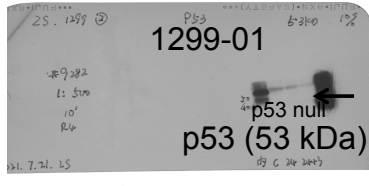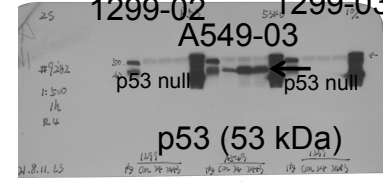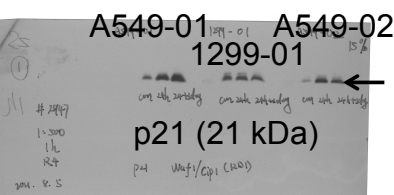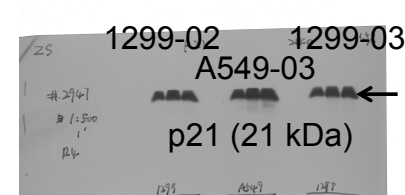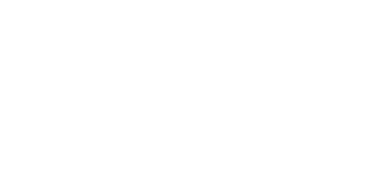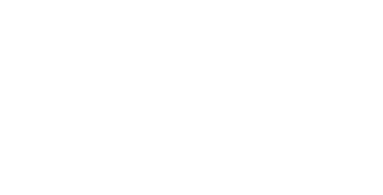

Supplement: Supplementary file 7 — Supplementary Information 7. [file 41598_2023_39880_MOESM7_ESM.pdf]
